# Surgical stabilization of serial rib fractures is advantageous in patients with relevant traumatic brain injury

**DOI:** 10.1007/s00068-022-01886-2

**Published:** 2022-02-07

**Authors:** Philipp Freitag, Cornelius Bechmann, Lars Eden, Rainer Meffert, Thorsten Walles

**Affiliations:** 1 Department of Cardiothoracic Surgery, Würzburg University Hospital, Josef-Schneider-Straße 2, 97080 Würzburg, Germany; 2 Department of Thoracic Surgery, Magdeburg University Medicine, Leipziger Strasse 44, 39120 Magdeburg, Germany; 3 Department of Trauma, Shoulder and Reconstructive Surgery, Rummelsberg Hospital, Rummelsberg 71, 90592 Schwarzenbruck, Germany; 4 Department of Trauma-, Hand-, Plastic- and Reconstructive Surgery, Würzburg University Hospital, Josef-Schneider-Straße 2, 97080 Würzburg, Germany

**Keywords:** Cerebral trauma, Clinical study, Cohort analysis, Polytrauma, Serial rib fracture, Surgical rib stabilization, Traumatic brain injury

## Abstract

**Purpose:**

To evaluate the clinical benefit of surgical stabilization of rib fractures (SSRF) in polytrauma patients with serial rib fractures.

**Methods:**

Retrospective single-center cohort analysis in trauma patients. Serial rib fracture was defined as three consecutive ribs confirmed by chest computer tomography (CT). Study cohort includes 243 patients that were treated conservatively and 34 patients that underwent SSRF. Demographic patient data, trauma mechanism, injury pattern, Injury Severity Score (ISS), Glasgow Coma Scale (GCS) and hospital course were analyzed. Two matched pair analyses stratified for ISS (32 pairs) and GCS (25 pairs) were performed.

**Results:**

The majority of patients was male (74%) and aged 55 ± 20 years. Serial rib fractures were associated with more than 6 broken ribs in average (6.3 ± 3.7). Other thoracic bone injury included sternum (18%), scapula (16%) and clavicula (13%). Visceral injury consisted of pneumothorax (51%), lung contusion (33%) and diaphragmatic rupture (2%). Average ISS was 22 ± 7.3. Overall hospital stay was 15.9 and ICU stay 7.4 days. In hospital, mortality was 13%. SSRF did not improve hospital course or postoperative complications in the complete study cohort. However, patients with a significantly reduced GCS (7.6 ± 5.3 vs 11.22 ± 4.8; *p* = 0.006) benefitted from SSRF. Matched pair analysis stratified for GCS showed shorter ICU stays (9 vs 15 days; *p* = 0.005) including shorter respirator time (143 vs 305 h; *p* = 0.003).

**Conclusion:**

Patients with serial rib fractures and simultaneous moderate or severe traumatic brain injury benefit from surgical stabilization of rib fractures.

## Introduction

Rib fractures are common in blunt chest trauma [1, 2]. While single rib fractures are accountable for high direct and indirect healthcare costs, multiple rib fractures result in significant patient morbidity and mortality due to chest wall instability and impaired respiratory mechanics [2–6]. As well patient as injury characteristics influence the clinical outcome after rib fractures: Increased age, a higher number of rib fractures, and presence of concomitant thoracic injuries are associated with poorer outcome including higher pneumonia rates, increased hospital and Intensive Care Unit (ICU) length of stay and increased mortality [2, 5–8]. In the last two decades, several medical implants have been introduced for rib osteosynthesis [9]. Technically, surgical stabilization of rib fractures (SSRF) goes along with supplementary soft tissue trauma to the injured chest wall and may additionally impair respiratory mechanics by muscle injury. As a result, no clear-cut selection criteria exist so far what patients benefit from SSRF [10–15]. Numerous studies have shown that early SSRF within the first week after chest trauma is effective to prevent secondary lung injury and to improve patient recovery [10, 12, 13]. In patients with multiple injuries chest trauma is the third most frequent cause of death after abdominal and traumatic brain injury (TBI). Moreover, simultaneous chest injury and TBI has a disastrous interdependency worsening patient prognosis [16]. SSRF to preserve or repair respiratory mechanics in polytrauma patients may, therefore, be a way to breach this vicious circle. But TBI treatment in polytrauma patients is a particular challenge for trauma surgeons and emergency physicians alike due to the imminent multifactorial risk of secondary brain injury and the tight time frame to avert additional brain injury and to improve patient prognosis [17–19]. Therefore, precipitative SSRF in polytrauma management may represent a preventable ‘second hit’ that aggravates patient recovery and prognosis [20, 21]. Well-founded decision making, therefore, is mandatory in this vulnerable patient cohort.

The aim of this study was to analyze the clinical benefit of SSRF in polytrauma patients the first two years after it was introduced at a German Level 1 trauma center.

## Patients and methods

### Study design

**Fig. 1:**
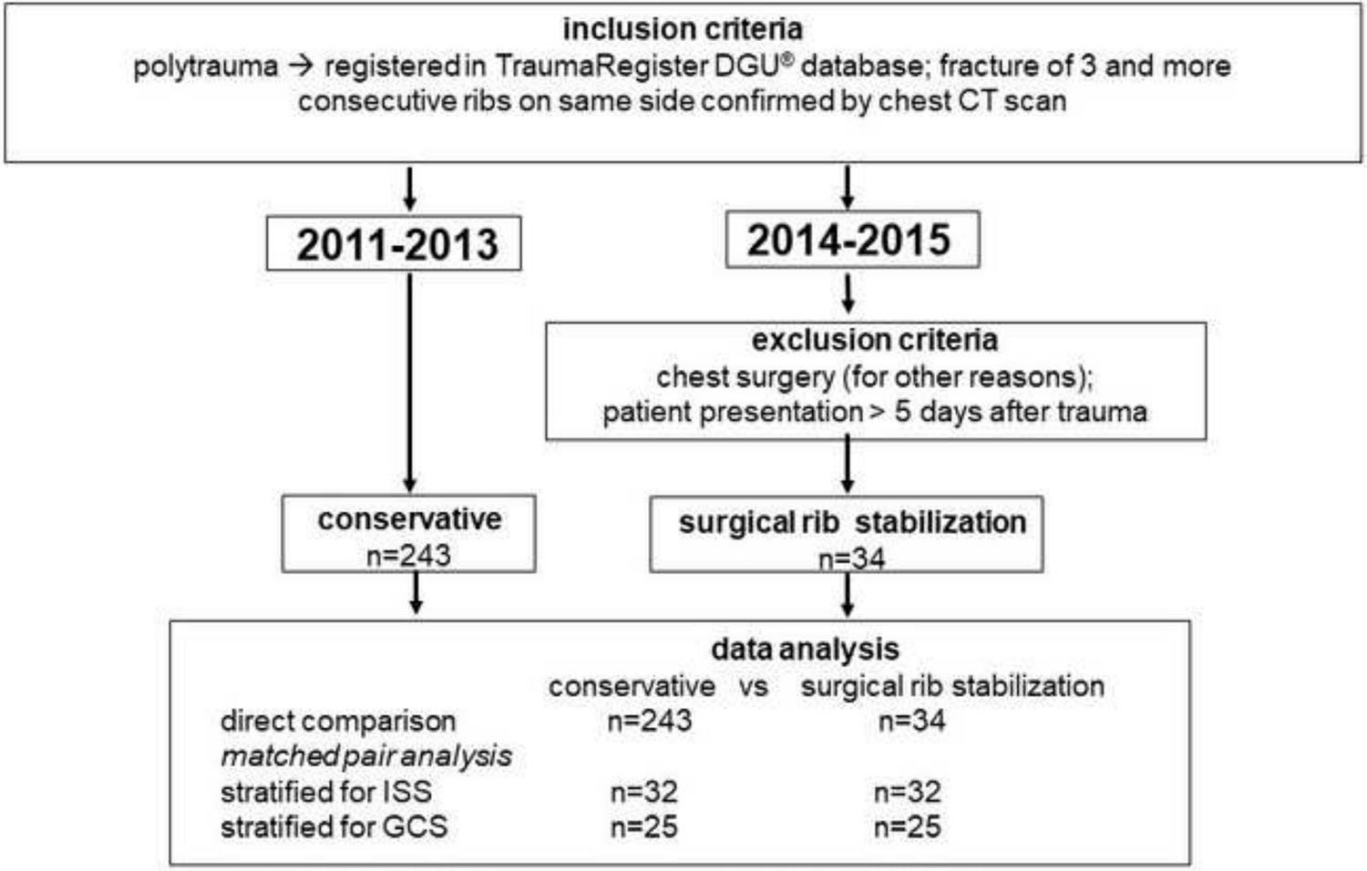
Study protocol. Records of polytrauma patients with serial rib fractures that were analyzed to determine the clinical effect of surgical rib stabilization. Patient cohorts were matched for ISS and GCS for data analysis (*ISS* injury severity score, *GCS* Glasgow coma scale)

SSRF was introduced at the trans-regional trauma center of the Würzburg University Hospital in 2014. Thenceforward the indication for SSRF was determined in multidisciplinary fashion among Trauma- and Thoracic Surgeons and Intensive Care Physicians. To evaluate the SSRF effect in polytrauma patients, all patients receiving SSRF for serial rib fractures in 2014 and 2015 were analyzed retrospectively (Fig. 1). The presence of serial rib fractures defined by 3 consecutive broken ribs on the same side was confirmed by re-evaluation of the computer-tomography (CT) of the chest at the time of hospital admission. To exclude selection bias, operated patients were compared to a historic cohort of patients treated between 2011 and 2013 that were matched to the operated patients according to Injury Severity Score (ISS) and Glasgow Coma Scale (GCS) [22, 23].

### Ethics approval

This retrospective chart review study involving human participants was in accordance with the ethical standards of the institutional and national research committee and with the 1964 Helsinki Declaration and its later amendments. Ethical approval was waived by the local Ethics Committee of Würzburg University Hospital in view of the retrospective nature of the study and all the procedures being performed were part of the routine care.

### Surgical indications for surgical rib stabilization

According to our institutional protocol surgical rib stabilization was offered to patients (1) with the presence of flail chest, (2) marked chest wall deformity, (3) serial rib fractures with poor pain control and (4) patients operated for other thoracic injuries. In case of multiple trauma, SSRF was offered according to prioritization for other injuries. Patients with extensive lung contusion, TRALI and ARDS were excluded. Surgical rib stabilization was considered only for the first week after trauma.

### Surgical technique

Operations were conducted under general anesthesia with double-lumen intubation. Patients were positioned in lateral decubitus position. To restore chest wall stability and respiratory mechanics, ribs 3–10 were approached. Far-flung multiple rib fractures of the same rib were repaired with two implants. The surgical focus was to restore chest wall stability and not every single broken rib was fixed. Skin and soft-tissue incision were performed directly above the area of rib fractures by a muscle-splitting approach to minimize vascular and neural injury. In the case of fracture lines underneath the scapula, the defect zone was approached by a longitudinal incision anterior or posterior of the scapula. The pleural cavity was inspected by video-assisted thoracoscopic surgery (VATS) for identification of additional intrathoracic injury and to clear residual hematothorax. A single 28 Ch chest tube was inserted for pleural drainage. Osteosynthesis was performed using a locked titanium plate fixation system (MatrixRIB™, DePuy Synthes, Umkirch, Deutschland). Depending on the costal defect, universal plates or pre-contoured plates were used and fixed with self-tapping locking screws. Implants were bended where necessary to fit each individual situation.

### Data collection

Data were retrieved from the institutional patient cohort that was reported to the national TraumaRegister DGU^®^ registry [24]. Demographic patient data, number and localization of rib fractures and concomitant chest injuries were analyzed. The Injury Severity Score and Glasgow Coma Scale at hospital admission were documented. To characterize the clinical outcome, ICU and hospital stay, length of mechanical ventilation, need for blood transfusions, rate of postoperative pneumonias and in-hospital mortality were analyzed.

### Statistical analysis

Discrete variables were expressed as counts and percentages and continuous variables as mean and standard deviation, unless stated otherwise. SSRF patients were compared to conservative treatment by matching gender, age (± 5 years), ISS score, and GCS score (“stratified for GCS”) or without GCS score (“stratified for ISS”). Comparison between groups was performed using Chi-squared test and *t* test for qualitative and quantitative data, respectively. All findings were considered statistically significant at *p* < 0.05. Analyses were performed using SPSS version 25.0 (SPSS Inc., Chicago, IL, USA).

## Results

Within the first two years after the implementation of surgical rib stabilization, 55 operations were performed in 227 polytrauma patients (ISS > 15; 24%). Of these patients, 34 patients (15%) fulfilled the inclusion criteria of this study. The operated patients were compared to similar patients that had been treated conservatively in the immediately previous time period.

### Baseline patient characteristics

The majority of patients was male and middle-aged (Table 1). Data analysis focused on trauma characteristics and pre-existing co-morbidities were not surveyed. Dominant accident causes were falls (37.9%) and motor vehicle accidents (32.9%). There were no differences between groups regarding pneumothorax rate, lung contusion, chest tube insertions and need for blood transfusions (*data not shown*). The number of broken ribs and concomitant clavicula fractures was higher in operated patients (6.3 ± 3.7 vs 8.4 ± 2.9 ribs, *p* = 0.001 and 12.8 vs 26.5%, *p* = 0.033). However, SSRF did not improve posttraumatic recovery or the need for intensive care medicine. In contrast, ventilation times and pneumonia rates increased after surgery, resulting in a significant extension of hospital stays (15.0 ± 10.7 vs 22.7 ± 15.5 days, *p* < 0.001) (Table 2). Mortality in non-operated patients was primarily due to respiratory failure (*n* = 13; 38%), shock (*n* = 11; 32%) and traumatic brain injury (*n* = 4; 12%). In patients selected for SSRF, shock was the only cause of death.

**Table 1:** Baseline data of patient cohorts

	Entire study cohort			Stratified for ISS			Stratified for GCS		
	Con	SSRF	*p*	Con	SSRF	*p*	Con	SSRF	*p*
Patient characteristics									
*n*	243	34		32	32		25	25	
Age (y)	55.5 ± 20.5	59.5 ± 13.5	0.279	60.47 ± 14.3	59.16 ± 13.5	0.706	57.7 ± 14.2	57.8 ± 13.7	0.984
Male (%)	73.7	76.5	0.727	78.1	78.1	1.000	88.0	88.0	1.000
ISS	19.6 ± 8.5	21.6 ± 9.0	0.211	22.0 ± 7.3	21.7 ± 9.1	0.892	21.8 ± 7.1	20.8 ± 7.6	0.647
GCS	12.3 ± 4.3	7.9 ± 5.3	< 0.001	11.2 ± 4.8	7.6 ± 5.3	0.006	7.9 ± 5.2	7.9 ± 5.4	1.000
Days after trauma (*n*)	–	3.4 ± 2.5		–	3.4 ± 2.5		–	3.2 ± 2.0	
Thoracic injury pattern									
Rib FX (*n*)	6.3 ± 3.7	8.4 ± 2.9	< 0.001	7.7 ± 2.6	8.3 ± 2.9	0.395	8.2 ± 3.5	8.2 ± 3.0	0.966
Scapula FX (%)	16.0	23.5	0.276	28.1	25.8	0.777	28.0	28.0	1.000
Clavicula FX (%)	12.8	26.5	0.033	9.4	28.1	0.550	16.0	24.0	0.724
Sternum FX (%)	17.7	17.6	0.994	25.0	18.8	0.454	12.0	20.0	0.700
Diaphragmatic injury (%)	0.8	11.8	< 0.001	0	12.5	0.039	0	16.0	0.037

*Con* conservative, *SSRF* surgical stabilization of rib fractures, *ISS* Injury Severity Score, *GCS* Glasgow Coma Scale, *FX* fracture

**Table 2:** Treatment results

	Entire study cohort			Stratified for ISS			Stratified for GCS		
	Con	SSRF	*p*	Con	SSRF	*p*	Con	SSRF	*p*
Hospital stay (d)	15.0 ± 10.7	22.7 ± 15.5	< 0.001	15.9 ± 13.0	23.4 ± 15.7	0.040	19.5 ± 11.5	20.9 ± 15.0	0.713
ICU stay (d)	8.3 ± 8.9	10.5 ± 7.8	0.175	9.6 ± 9.4	10.8 ± 7.9	0.623	15.4 ± 8.4	8.9 ± 7.2	0.005
Mechanical ventilation (h)	118 ± 193	172 ± 184	0.127	153 ± 204	183 ± 184	0.546	305 ± 195	143 ± 161	0.003
Pneumonia rate (%)	4.5	14.7	0.017	3.1	12.9	0.162	12	12	1.000
Mortality (%)	13.2	2.9	0.085	15.6	3.2	0.086	12.0	4.0	0.297

*Con* conservative, *SSRF* surgical stabilization of rib fractures, *ISS* injury severity score, *GCS* Glasgow coma scale, *ICU* intensive care unit

### Effect of trauma severity

Although the number of rib fractures was significantly increased in operated patients, both study groups did not differ in overall severity of the cumulative trauma as indicated by the ISS (19.6 ± 8.5 vs 21.6 ± 9.0, *p* = 0.211). Accordingly, the ISS was not helpful to indicate patients that would benefit from SSRF.

### Effect of concomitant brain injury

The indication for SSRF was made within the early post-traumatic phase on the basis of impaired chest wall stability and respiratory mechanics. Our retrospective analysis of operated patients revealed that the SSRF cohort showed a striking increased neurological impairment (GCS 12.3 ± 4.3 vs 7.9 ± 5.3, *p* < 0.001) (Table 1). Therefore we conducted a matched-pair analysis of all patients with a relevant neurological impairment. This analysis documented a distinct prognostic advantage for operated patients following SSRF: Patients benefit from halving of artificial ventilation times (143 ± 161 vs 305 ± 195 h, *p* = 0.003) resulting in shorter ICU stays (8.9 ± 7.2 vs 15.4 ± 8.4 days, *p* = 0.005) (Table 2). Pneumonia rates do not vary between operated and non-operated patients. Mortality drops from 12 to 4% without reaching statistical significance in our analysis.

## Discussion

Numerous previous studies have shown that SSRF is beneficial and safe in patients with serial rib fractures and flail chest [2, 5–8, 10, 15, 25]. Ideally, patients are operated on within the first week after trauma to prevent pneumonia and respiratory failure requiring mechanical ventilation [12, 13, 15, 25, 26]. Currently, no consensus exists for SSRF indications in patients with multiple injuries and polytrauma [13, 21, 27].

This study in polytrauma patients showed that SSRF is beneficial only in a fraction of patients. Although the indication to offer SSRF based on the clinical presence of chest wall instability in patients with confirmed serial rib fractures, SSRF increased the need for intensive care support including mechanical ventilation in our study cohort. Especially, in our retrospective analysis we observed a significant increase in pneumonia rates in patients undergoing SSRF. Our retrospective analysis illustrates that patients with more severe thoracic injury patterns were selected for SSRF as can be conveyed by the number of broken ribs (Table 1). However, this increased trauma burden is not reflected in the ISS score of our patients. This selection bias may explain the deteriorated clinical course of operated patients compared to conservative treatment. In what way this observation can additionally be attributed to ‘second hit’ effects on biochemical and physiologic alterations occurring in polytrauma patients undergoing SSRF remains speculative and cannot be answered by our analysis of clinical data [28].

A more detailed evaluation of our two patient cohorts revealed that although they were similar regarding the sustained injuries, the operated patients showed a significant impairment of their neurocognitive function. Therefore, we matched non-operated patients and patients undergoing SSRF according to their GCS. This analysis is a very small patient number revealed that this subgroup of patients significantly benefitted from SSRF by halving ventilator dependency resulting in shorter ICU stays. Moreover, patient mortality was reduced from 12 to 4% without reaching statistical significance. This effect can be attributed in part to SSRF since no operated patient died because of respiratory failure. Traumatic shock was the leading cause of death in this group. Interestingly, the interdisciplinary clinical selection of patients for SSRF successfully excluded those patients that deceased due to secondary traumatic brain injuries [17, 28, 29]. The discriminator for polytrauma patients with concomitant traumatic brain injury benefitting from SSRF was a GCS of about 8 in our study.

Our finding challenges the general opinion that SSRF is not warranted in patients with mechanical ventilation due to cerebral injury [13]. However, pulmonary recovery after trauma is driven by both chest wall stability and patient cooperation in physiotherapy to restore broncho-pulmonal clearance including coughing-ability. We hypothesize that the latter is significantly delayed in patients with cerebral injury and that these patients benefit from the surgical restoration of chest wall stability. Our observation is supported by another retrospective single-center cohort analysis [30].

A secondary finding in our operated patient cohort is that more than 10% of patients with serial rib fractures sustain diaphragmatic injuries that are not detected by chest-CT at hospital admission. The diaphragmatic injuries represent lacerations or small perforations and not diaphragmatic ruptures with the risk of organ incarceration or displacement. The patients’ clinical courses confirm that the impact of those injuries is negligible.

Our study is limited by its mono-centric nature with small patient numbers and its retrospective data analysis. In addition, our analysis did not investigate a possible role of pre-existing co-morbidities in our trauma patients. However, the comparison of operated patients with a historic patient cohort from the very same center obviates a selection bias that is present in any other study design except prospective randomized controlled clinical trials. The latter are hardly realizable in the acute trauma setting [14].

## Conclusion

SSRF in polytrauma patients with serial rib fractures is advantageous in patients with moderate and severe TBI. We suggest SSRF within the first week after trauma in patients (1) with the presence of flail chest, (2) marked chest wall deformity, (3) serial rib fractures with poor pain control and (4) patients operated for other thoracic injuries. Interdisciplinary counselling is crucial to exclude patients deteriorating from secondary brain injury. SSRF showed no benefit in polytrauma patients with only mild TBI. Given the small patient number in this analysis our findings should be reappraised in future multi-centric analyses with more treatments.

## Data Availability

Not applicable.

## Abbreviations

ARDS: Acute respiratory distress syndrome
Ch: Charrière (= french gauge system)
CT: Computer-tomography
DGU: German Society for Traumatology
GCS: Glasgow Coma Scale
ICU: Intensive Care Unit
ISS: Injury severity score
SSRF: Surgical stabilization of rib fractures
TBI: Traumatic brain injury
TRALI: Transfusion-related acute lung injury
VATS: Video-assisted thoracoscopic surgery

## References

[1] Hoefer C, Lefering R. Annual report 2020—Traumaregister DGU. In: TraumaRegister DGU^®^. www.auc-online.de. 2020. Accessed 24. April 2021.

[2] Prins JT, Wijffels MM, Wooldrik SM, Panneman MJ, Verhofstad MH, Van Lieshout EM (2021). Trends in incidence rate, health care use, and costs due to rib fractures in the Netherlands. Eur J Trauma Emerg Surg.

[3] Flagel BT, Luchette FA, Reed RL, Esposito TJ, Davis KA, Santaniello JM, Gamelli RL (2005). Half-a-dozen ribs: the breakpoint for mortality. Surgery.

[4] Kent R, Woods W, Bostrom O. Fatality risk and the presence of rib fractures. In: annals of advances in automotive medicine/annual scientific conference 2008. Association for the advancement of automotive medicine. PMID: 19026224. PMC3256783 19026224

[5] Abdulrahman H, Afifi I, El-Menyar A, Al-Hassani A, Almadani A, Al-Thani H, Latifi R (2013). Clinical outcomes of multiple rib fractures: does age matter?. Eur J Trauma Emerg.

[6] El-Menyar A, Latifi R, AbdulRahman H, Zarour A, Tuma M, Parchani A, Peralta R, Al TH (2013). Age and traumatic chest injury: a 3-year observational study. Eur J Traum Emerg.

[7] Bulger EM, Arneson MA, Mock CN, Jurkovich GJ (2000). Rib fractures in the elderly. J Trauma.

[8] Van Vledder MG, Kwakernaak V, Hagenaars T, Van Lieshout EMM, Verhofstad MHJ, South West Netherlands Trauma Region Study G (2019). Patterns of injury and outcomes in the elderly patient with rib fractures: a multicenter observational study. Eur J Trauma Emerg Surg.

[9] Bemelmann M. Rib fixation: old wine in new bottles? In: CTSNet. 2014. https://www.ctsnet.org/article/rib-fixation-old-wine-new-bottles. Accessed 24 Apr 2021

[10] Olsen MF, Pazooki D, Granhed H (2013). Recovery after stablising surgery for ‘flail chest’. Eur J Trauma Emerg.

[11] Said SM, Goussous N, Zielinski MD, Schiller HJ, Kim BD (2014). Surgical stabilization of flail chest: the impact on postoperative pulmonary function. Eur J Trauma Emerg.

[12] Pieracci FM, Coleman J, Ali-Osman F, Mangram A, Majercik S, White TW, Jeremitsky E, Doben AR (2018). A multicenter evaluation of the optimal timing of surgical stabilization of rib fractures. J Trauma Acute Care Surg.

[13] Olland A, Puyraveau M, Guinard S, Seitlinger J, Kadoche D, Perrier S, Renaud S, Falcoz PE, Massard G (2019). Surgical stabilization for multiple rib fractures: whom the benefit?—a prospective observational study. J Thorac Dis.

[14] Beks RB, de Jong MB, Sweet A, Peek J, van Wageningen B, Tromp T, IJpma F, Wouters R, Lansink K, Bemelman M, van Baal M (2019). Multicentre prospective cohort study of nonoperative versus operative treatment for flail chest and multiple rib fractures after blunt thoracic trauma: study protocol. BMJ Open.

[15] Peek J, Beks RB, Hietbrink F, Heng M, De Jong MB, Beeres FJP, Leenen LPH, Groenwols RHH, Houwert RM (2020). Complications and outcome after rib fracture fixation: a systematic review. J Trauma Acute Care Surg.

[16] Hofman M, Andruszkow H, Kobbe P, Poeze M, Hildebrand F (2020). Incidence of post-traumatic pneumonia in poly-traumatized patients: identifying the role of traumatic brain injury and chest trauma. Eur J Trauma Emerg Surg.

[17] Rickels E (2017). Focus on traumatic brain injury. Eur J Trauma Emerg Surg.

[18] Geeraerts T, Velly L, Abdennour L, Asehnoune K, Audibert G, Bouzat P, Bruder N, Carrillon R, Cottenceau V, Cotton F, Courtil-Teyssedre S, Dahyot-Fizelier C, Dailler F, David JS, Engrand N, Fletcher D, Francony G, Gergelé L, Ichai C, Javouhey É, Leblanc PE, Lieutaud T, Meyer P, Mirek S, Orliaguet G, Proust F, Quintard H, Ract C, Srairi M, Tazarourte K, Vigué B, Payen JF, French Society of Anaesthesia; Intensive Care Medicine; in partnership with Association de neuro-anesthésie-réanimation de langue française (Anarlf); French Society of Emergency Medicine (Société Française de Médecine d'urgence (SFMU); Société française de neurochirurgie (SFN); Groupe francophone de réanimation et d’urgences pédiatriques (GFRUP); Association des anesthésistes-réanimateurs pédiatriques d’expression française (Adarpef) (2018). Management of severe traumatic brain injury (first 24hours). Anaesth Crit Care Pain Med.

[19] Thapa K, Khan H, Singh TG, Kaur A (2021). Traumatic brain injury: mechanistic insight on pathophysiology and potential therapeutic targets. J Mol Neurosci.

[20] Ball CG (2015). Damage control surgery. Curr opin Crit Care.

[21] Molnar TF (2019). Thoracic damage control surgery. J Thorac Dis.

[22] Linn S (1995). The injury severity score—importance and uses. Ann Epidemiol.

[23] Green SM, Haukoos JS, Schriger DL (2017). How to measure the glasgow coma scale. Ann emerg Med.

[24] AUC – Akademie der Unfallchirurgie GmbH. Traumaregister DGU^®^. https://traumaregister.auc-online.de. Accessed 24. April 2021

[25] Wijffels MME, Hegenaars T, Latifi D, Van Lieshout EMM, Vorhofstad MHJ (2000). Early results after operative versus non-operatively treated flail chest: a retrospective study focusing on outcome and complications. Eur J Trauma Emerg Surg.

[26] Lehmann M, Oehler B, Zuber J, Malzahn U, Walles T, Muellenbach RM, Roewer N, Kredel M (2020). Redistribution of pulmonary ventilation after lung surgery detected with electrical impedance tomography. Acta Anaesthesiol Scand.

[27] Chrysou K, Halat G, Hoksch B, Schmid RA, Kocher GJ (2017). Lessons from a large trauma center: impact of blunt chest trauma in polytrauma patients-still a relevant problem?. Scand J Trauma Resusc Emerg Med.

[28] Lasanianos NG, Kanakaris NK, Dimitriou R, Pape HC, Giannoudis PV (2011). Second hit phenomenon: existing evidence of clinical implications. Injury.

[29] McCredie VA, Chavarría J (2021). Baker AJ How do we identify the crashing traumatic brain injury patient—the intensivist's view. Curr Opin Crit Care.

[30] Kocher GJ, Sharafi S, Azenha LF, Schmid RA (2017). Chest wall stabilization in ventilator-dependent traumatic flail chest patients: who benefits?. Eur J Cardiothorac Surg.

